# Feature Mining and Health Assessment for Gearboxes Using Run-Up/Coast-Down Signals

**DOI:** 10.3390/s16111837

**Published:** 2016-11-02

**Authors:** Ming Zhao, Jing Lin, Yonghao Miao, Xiaoqiang Xu

**Affiliations:** 1 School of Mechanical Engineering, Xi’an Jiaotong University, Xi’an 710049, China; zhaomingxjtu@mail.xjtu.edu.cn (M.Z.); miaoyonghao@stu.xjtu.edu.cn (Y.M.); xuxiaoqiang@stu.xjtu.edu.cn (X.X.); 2 State Key Laboratory for Manufacturing Systems Engineering, Xi’an Jiaotong University, Xi’an 710049, China

**Keywords:** generalized phase demodulation, run-up/coast-down analysis, feature mining and integration, gearbox health assessment, phasogram

## Abstract

Vibration signals measured in the run-up/coast-down (R/C) processes usually carry rich information about the health status of machinery. However, a major challenge in R/C signals analysis lies in how to exploit more diagnostic information, and how this information could be properly integrated to achieve a more reliable maintenance decision. Aiming at this problem, a framework of R/C signals analysis is presented for the health assessment of gearbox. In the proposed methodology, we first investigate the data preprocessing and feature selection issues for R/C signals. Based on that, a sparsity-guided feature enhancement scheme is then proposed to extract the weak phase jitter associated with gear defect. In order for an effective feature mining and integration under R/C, a generalized phase demodulation technique is further established to reveal the evolution of modulation feature with operating speed and rotation angle. The experimental results indicate that the proposed methodology could not only detect the presence of gear damage, but also offer a novel insight into the dynamic behavior of gearbox.

## 1. Introduction

Vibration analysis has been widely accepted and extensively used for the health assessment and fault diagnosis of rotating machinery. Many efforts have been made to develop accurate diagnostic methods based on vibration analysis over the past few decades [1,2,3,4]. Most of those methods, however, are built on a basic assumption that the machinery being measured is running under stationary speeds. With the advances of modern industry, more and more machines, ranging from large-scale equipment like wind turbines and mine excavators, to some key components such as high speed spindles and vehicle bearings, are operating under variable speed conditions (VSC). The variation of speed will bring complicated amplitude- and frequency-modulation effects to the measured signal. In many cases, those speed-related modulation effects are much more powerful than those caused by mechanical defects, thus imposing great difficulty for fault detection and health assessment. 

Confronted with those challenges, the condition monitoring of machinery under VSC is gaining increasing attention in both industry and academia [5,6,7,8,9]. A number of research papers, technical reports, and conference proceedings have published on this topic in the last five years. A specific international conference called CMMNO (Condition Monitoring of Machinery in Non-Stationary Operations) has been organized since 2011 to analyze the research challenges we face, and also propose some methodologies to deal with this issue. Generally, recent advances in this direction can be classified into two groups. Since the signal measured in this condition is non-stationary in nature, the first group resorts to some non-stationary signal analysis tools such as short-time Fourier transform (STFT), empirical mode decomposition (EMD) [10,11], wavelet, chirplet, synchro-squeezing transform [12,13] and, more recently, proposed dynamic time warping. For instance, Meltzer et al. [14] proposed a polar wavelet amplitude map to realize the fault diagnosis of gears operating under non-stationary rotation speeds. Zhao et al. [15] employed Chirplet transform to extract and enhance the time-varying meshing frequency and its sidebands associated with gear defect. A generalized synchro-squeezing transform was further proposed by Li et al. [16,17] for the gear fault detection and diagnosis. In this approach, a time-scale restoration operation is initially presented to obtain a concentrated time-frequency representation (TFR), the gear defect can then be identified by observing the gear meshing frequency and its sidebands in the TFR. To avoid the spectral leakage and aliasing problem associated with Fourier transform, a dynamic time warping (DTW) method is introduced by Zhen et al. [18,19] to detect the fault in a two-stage reciprocating compressor under different operating conditions. More recently, Liu et al. [20,21] further extended this method to the gearbox diagnosis by the jointly use of fast DTW and correlated kurtosis. Though the above methods have shown their effectiveness for signal analysis and fault detection under VSC, the high computational complexity and long running time are the major limitations in real applications. 

Due to the symmetric mechanical structure, the vibration signal of a rotating machine is essentially periodic in angle, rather than in time. Consequently, the non-stationary phenomena caused by speed variation can be largely removed if the signal is represented in angular domain. Benefitting from that, the second group first utilizes order tracking technique to transform the raw signal from time domain to angular domain, and then treats it as a conventional constant speed problem [8,22]. However, it should be stressed that the signals measured under VSC is inherently more informative than these in constant speed. Therefore, it is believed that the prospects for VSC analysis should not be limited to removing the barriers caused by speed variation, but rather gain some new insight into the machine behavior under VSC or, exploit some crucial fault phenomena that easily be ignored under constant speed. Thus far, however, few investigations have been conducted toward this direction.

In view of this research gap, a systematic methodology for VSC signal analysis is proposed in this work, and particular emphasis is placed on the run-up/coast-down (R/C) signal of a gearbox which is a typical VSC scenario that is frequently encountered in many industrial applications. In the proposed methodology, we first investigate the data preprocessing and feature selection issues for gearbox. Built on that, a sparsity-guided feature enhancement scheme is proposed to extract the weak phase jitter associated with gear fault. In order for an effective feature mining and integration under R/C, a generalized phase demodulation technique is finally established to reveal the evolution of fault feature with operating speed and rotation angle. The experimental results indicate that the proposed methodology could not only detect the presence of gear damage, but also offer a novel insight into the dynamic behavior of gearbox. The rest of this paper is organized as follows. The vibration model of gearbox in the R/C process is introduced in Section 2. The principle and implementation of the proposed method are elaborated in Section 3. The performance of the proposed method is validated by the coast down data measured from a two-stage gearbox in Section 4. Finally, some conclusions are drawn in Section 5.

## 2. Vibration Model for Gearbox in the R/C Process

A proper description and understanding of vibration behavior of gearbox is key to the feature selection and feature mining. For this reason, this section will begin with a basic vibration model of a healthy gearbox under constant speed, and then it is extended to a more complicated scenario, i.e., a defective gearbox in the R/C processes.

Generally, the vibration signals measured from a healthy gear gearbox mainly consist of gear meshing frequency (GMF) and its harmonics, which can be given as [23]

(1) $$x(t)=\sum_{k=1}^{K}X_{k}\cos\varphi_{k}(t)=\sum_{k=1}^{K}X_{k}\cos(2\pi kTft+\Phi_{k})$$

where *K* denotes the number of gear meshing harmonics; *T* is the number of gear teeth; *f* represents the rotating frequency of gear shaft; *φ_k_*(*t*) stands for the instantaneous phase of the *k*th meshing harmonic; and *X_k_* and Φ*_k_* are its amplitude and initial phase, respectively. 

When a local defect occurs, the contact stiffness, bending stiffness as well as the shear stiffness of gear will change as the faulty tooth engages into mesh [24,25]. This in turn produces amplitude and phase modulation to the vibration signal. As those modulation effects are periodic with shaft rotating frequency *f*, they can be represented by the following Fourier series form

(2) $$\begin{array}{l} a_{k}(t)=\sum_{n=0}^{N}A_{kn}\cos(2\pi nft+\alpha_{kn})\\ b_{k}(t)=\sum_{n=0}^{N}B_{kn}\cos(2\pi nft+\beta_{kn}) \end{array}$$

where *N* is the number of sidebands around gear meshing harmonics; and *A_kn_* and *α_kn_* are, respectively, the amplitude and phase of the *n*th sideband of *a_k_*(*t*) around the *k*th meshing harmonic, while *B_kn_* and *β_kn_* are those of *b_k_*(*t*) around the *k*th meshing harmonic.

Taking the above modulation effects into account, the vibration signal of a faulty gear can therefore be represented by

(3) $$x(t)=\sum_{k=1}^{K}X_{k}[1+a_{k}(t)]\cos[2\pi kTft+\Phi_{k}+b_{k}(t)].$$

**Figure 1:**
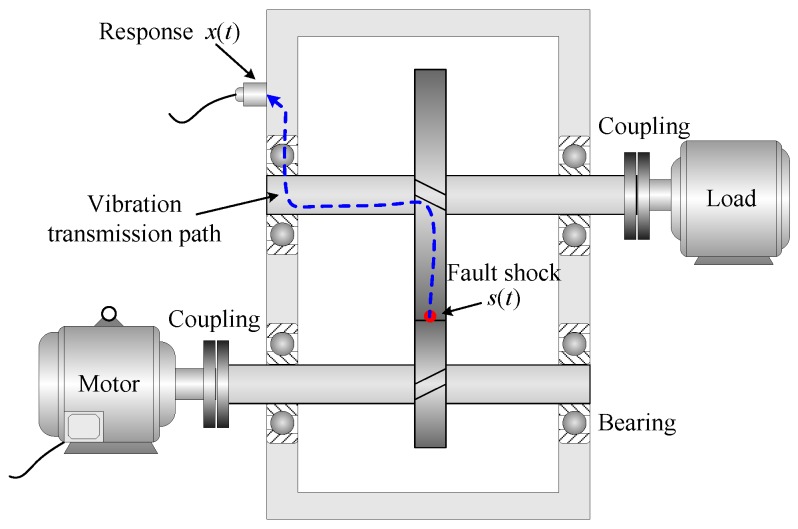
Transfer path of vibration signal.

Note that the amplitude modulation *a_k_*(*t*) and phase modulation *b_k_*(*t*) carry rich information regarding the health status of gearbox. When the gearbox operates under a constant speed and load, those modulations are manifested as equally spaced sidebands around the GMF and its harmonics in the spectrum. In this case, *a_k_*(*t*) and *b_k_*(*t*) can easily be obtained by means of synchronous averaging and classical demodulation technique. When the gearbox undergoes a R/C process, however, the extraction of amplitude and phase modulation is no longer an easy task. Firstly, the constantly varying speed will give rise to spectral smearing phenomenon, in which the GMF and its sidebands are blended together in the spectrum. Therefore, some signal preprocessing procedures are required before the application of more advanced diagnostic approaches. Secondly, there is always a long transmission from a defect to a transducer in applications [26,27]. Taking Figure 1 as an example, the gear vibration will successively transmit through the gear disk, the shaft, the bearing, the gearbox casing, and finally arrives at the vibration transducer. As a result, the measured signal is inevitably affected by the transmission path effect. If the path is simplified as a linear cascade system, the relationship between fault shock *s*(*t*) and its vibration response *x*(*t*) can be given by Equation (4) in frequency domain [28]

(4) $$X(f)=H(f)\cdot S(f)=H_{c}(f)\cdot H_{b}(f)\cdot H_{s}(f)\cdot H_{g}(f)\cdot S(f)$$

where *X*(*f*) and *S*(*f*) denote the Fourier transforms of *x*(*t*) and *s*(*t*), respectively. H(f) is the frequency response (FR) of the whole transfer path, which is the product of the individual influence from gear $H_{g}(f)$, shaft $H_{s}(f)$, bearing $H_{b}(f)$ and casing $H_{c}(f)$. 

Since the FR of transfer path is a function of *f*, the fault response as well as its modulation feature would vary with the rotation frequency of gearbox. To account for this effect, an independent variable *f* is added to Equation (3), and the vibration model for gearbox in the R/C process can be given as

(5) $$x(f,t)=\sum_{k=1}^{K}X_{k}(f)\cdot [1+{\tilde{a}}_{k}(f,t)]\cos[2\pi kTft+\Phi_{k}+{\tilde{b}}_{k}(f,t)]$$

where ${\tilde{a}}_{k}(f,t)$ and ${\tilde{b}}_{k}(f,t)$ are the modified amplitude and phase modulations, respectively. 

**Figure 2:**
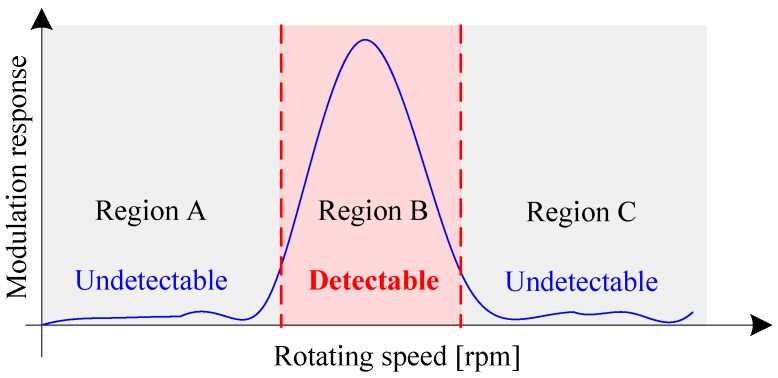
Speed-dependent modulation characteristic of gearbox.

From Equation (5), one can infer that, as speed changes, the modulation effect induced by fault would be magnified or suppressed as illustrated in Figure 2. Therefore, when the test speed of gearbox is not properly selected (for instance, in Region A or Region C), the fault feature would be very weak and easily be masked by background noise. In that case, the gear fault is undetectable. In fact, this speed-dependent modulation characteristic (SDMC) is a crucial feature of gearbox, because it contains not only the health information of gearbox but also the optimal testing speed under which the fault feature is fully highlighted. In theory, the SDMC could be obtained by finite element analysis (FEA). However, FEA may encounter several difficulties in practice. Firstly, FEA is based on some simplified model, and it is only effective when certain underlying assumptions hold. For instance, it is rather difficult to simulate and analyze the non-linear dynamics of oil film between the meshing gears. Secondly, FEA can hardly consider the influences of manufacturing and installation errors on the vibration response of gearbox. In fact, even for the same type of gearboxes, the vibration may differ vastly due to different installation and manufacturing conditions. Thirdly, FEA cannot simulate the real operating condition of gearbox accurately. It is worth mentioning that the variable speed and dynamic load are also key factors that influence the vibration behavior of gearbox. For these reasons, the experimental extraction of SDMC using the R/C signals is of great interest.

## 3. Feature Mining and Health Assessment of Gearbox using R/C Signals

Based on the above signal modeling and modulation analysis, a novel framework, which consists of signal preprocessing, feature enhancement, SDMC mining and integration, is proposed for the health assessment of gearbox. The principle and implementation of the method are elaborated as follows.

### 3.1. Signal Preprocessing using Computed Order Tracking

As discussed in the previous section, the fault information of gear is mainly embedded in the modulation sidebands. However, those sidebands are aliased together as a result of large speed variation in the R/C process. 

**Figure 3:**
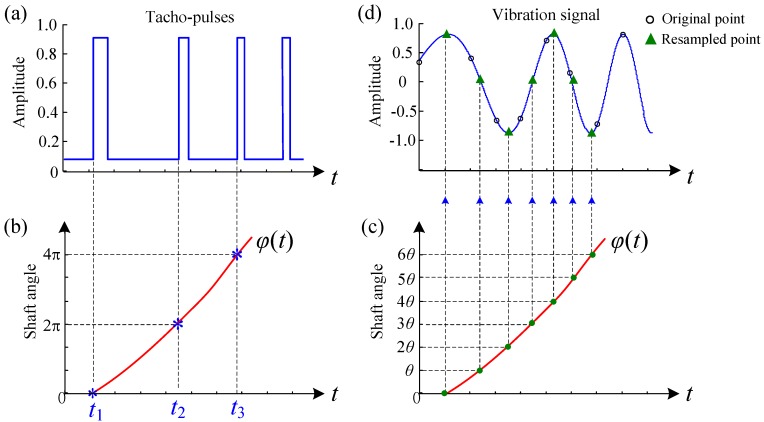
Implementation of COT: (**a**) determine the pulses arrive times; (**b**) obtain the *φ*(*t*) by fitting; (**c**) calculate the resampling time instants for the constant angular increments *θ*; and (**d**) signal resampling by interpolation.

Order tracking is an effective tool to process vibration signals measured under variable speed conditions [29,30,31,32,33]. In this technique, the vibration signal with constant time intervals is digitally resampled at constant increments of shaft angle. Since the vibration signal is approximately periodic in the angular domain, the spectral smearing problem can be eliminated thereafter. In practice, order tracking could be implemented in three different ways, namely, hardware based order tracking (HOT), computed order tracking (COT) [30,34] and phase demodulation based order tracking (PDOT) [15,22,35,36]. Each of these methods has its pros and cons, as discussed in Ref. [28]. In this work, COT is employed due to its high-reliability and easy-to-implement. Since COT is well established, we only give a graphically introduction here, as shown in Figure 3. For more details of COT, refer to [30,34]. 

### 3.2. Feature Selection and Enhancement: A Sparsity-Guided Phase Jitter Sharpening Scheme

After COT, the smeared meshing component and its sidebands could be recovered in the spectrum of resampled signal, which is also known as order spectrum. According to the vibration model given in Section 2, both the amplitude modulation and phase modulation can be utilized to assess the health status of gearbox. In practice, however, the phase modulation was found to be more effective for detecting incipient faults of gears, and in general yields better results than amplitude modulation [37]. For this reason, it is selected as the target feature in this work. 

**Figure 4:**
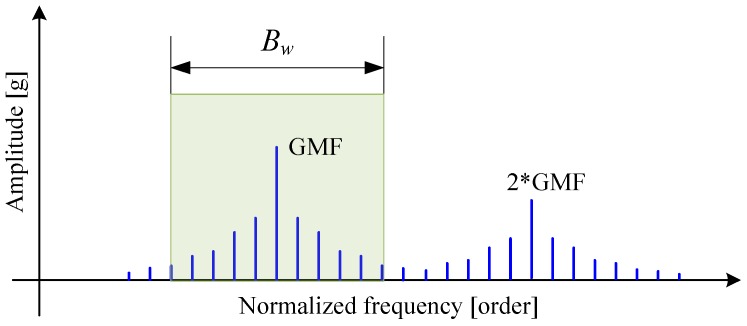
Sidebands extraction using band-pass filtering.

**Figure 5:**
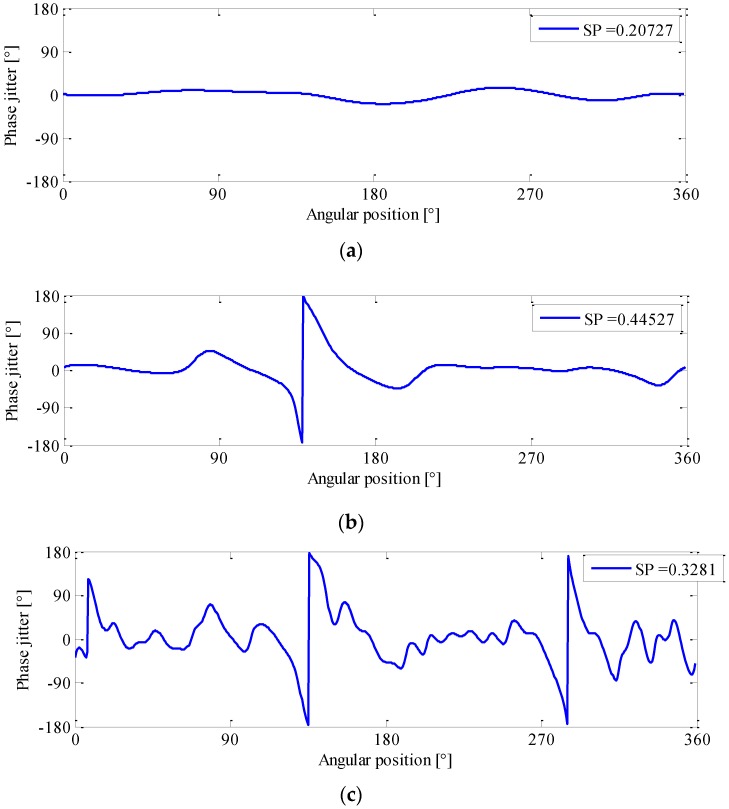
Phase demodulation results and their corresponding sparsity parameter (SP) for: (**a**) *B_w_* = 4 order; (**b**) *B_w_* = 8 order; and (**c**) *B_w_* = 16 order.

To perform phase demodulation, a number of sidebands first need to be extracted from the order spectrum using band-pass filtering, as illustrated in Figure 4. In order for a better performance of phase demodulation, the filter bandwidth should be carefully chosen. Figure 5 exhibits a typical example of phase demodulation results using different bandwidths. It can be seen from Figure 5a that, if the bandwidth were too narrow, the phase jitter caused by fault would be smoothed, in which the gear fault can easily be overlooked. In contrast, if it were too wide, much more noise will be included in the filtered signal, thus producing many spurious peaks as illustrated in Figure 5c. In current applications, the filter bandwidth is commonly determined by trial and error. Obviously, such a determination manner is not suitable for the analysis of R/C signal because, similar to its modulation behavior, the optimal bandwidth is also speed dependent. For this reason, a data-driven bandwidth selection scheme is quite necessary. 

By revisiting Figure 5, one may find that a proper filter bandwidth selection could effectively capture the local discontinuity in meshing, and thereby yielding a shape phase jitter in the demodulated signal as shown in Figure 5b. In fact, this local energy concentration of signal can be well depicted by a statistical indicator termed sparsity.

In the community of signal processing, sparsity is used to describe the energy distribution of a signal [38]. Specifically, the sparsity of a signal is 1 if all its energy is concentrated in only one element (perfect sparse). On the contrary, if the energy is evenly distributed among all elements, its sparsity is zero (non-sparse at all). Till now, a number of sparsity measures have been proposed in the literature, including *l*_2_/*l*_1_ norm, *pq*-mean, Hoyer measure, and Gini index. In the field of mechanical signal processing, *l*_2_/*l*_1_ norm has been successfully used for optimal demodulation band selection [39], wavelet parameter optimization [40], ultrasonic signal deconvolution [41], etc. To compare the performance of different sparse measures, an in-depth investigation was conducted by Hurley [38]. It is found that the *l*_2_/*l*_1_ norm could perform better and would have more nice properties after normalization [38]. For this reason, the normalized *l*_2_/*l*_1_ norm, also termed as Hoyer measure [42], is adopted in this work. The Hoyer measure is mathematically defined as

(6) $$SP(x)=\frac{\sqrt{n}-l_{1}(x)/l_{2}(x)}{\sqrt{n}-1}=\frac{\sqrt{n}-\left(\sum \left|x_{i}\right|\right)/\sqrt{\sum x_{i}^{2}}}{\sqrt{n}-1}$$

where *l*_1_(**x**) and *l*_2_(**x**) are, respectively, the *l*_1_ and *l*_2_ norm of signal **x**, and *n* is its length. 

Using this measure, the sparseness of the phase demodulated signals in Figure 5 are evaluated, and their sparsity values are 0.21, 0.45, and 0.33, respectively. Note that if the bandwidth is proper selected, the phase jitter can be effectively enhanced and accounts for more energy of the demodulated signal, which in turn increases the sparsity value. On the contrary, the sparsity decreases as the energy is more evenly distributed. Based on this idea, we attempt to automate the bandwidth selection by maximizing the sparsity value of demodulated signal, which can be formulated as

(7) $${\bar{B}}_{w}=\underset{B_{w}}{\arg\max}\left\{SP({\tilde{b}}_{k}^{B_{w}}(f,t))\right\}$$

where ${\tilde{b}}_{k}^{B_{w}}(f,t)$ denotes the phase demodulated signal around *k*th meshing harmonic with bandwidth of *B_w_*.

### 3.3. Feature Mining and Integration: A Generalized Phase Demodulation Technique

As we know, the R/C signals usually carry abundant information about the health status of gearbox. However, a key issue is how could this information be effectively exploited and utilized to achieve a reliable health assessment result. In this subsection, a generalized phase demodulation (GPD) technique is proposed for feature mining and integration.

**Figure 6:**
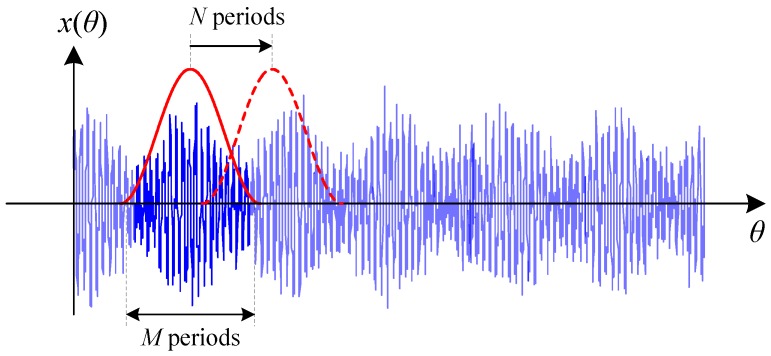
Illustration of signal segmentation procedure.

Inspired by STFT, the GPD first divides the resampled R/C data into short segments using a sliding window as illustrated in Figure 6. Each segment comprises exactly *M* revolution of vibration signals of the interested gear. To ensure all the segments have the same initial phase (or angular position), the sliding step of the window is also specified as an integer gear periods denoted by *N*. Typically, *N* is selected less than *M* in applications, which means the consecutive segments could overlap with each other.

Since the vibration signal has already been order tracked, synchronous averaging can be directly performed on each segment to remove the non-synchronous components and measurement noise. After that, the optimal bandwidth for each segment is determined by the sparsity-guided scheme as given above. Using this parameter, the narrowband signal around the *k*th meshing harmonic can then be obtained as

(8) $$y(f_{i},\theta )=X_{k}(f_{i})[1+{\tilde{a}}_{k}^{B_{wi}}(f_{i},\theta )]\cos[j(kT\theta +\Phi_{k}+{\tilde{b}}_{k}^{B_{wi}}(f_{i},\theta ))]$$

By Hilbert transform, the analytical form of the narrow-band modulation signal is given by

(9) $$z(f_{i},\theta )=X_{k}(f_{i})[1+{\tilde{a}}_{k}^{B_{wi}}(f_{i},\theta )]\exp[j(kT\theta +\Phi_{k}+{\tilde{b}}_{k}^{B_{wi}}(f_{i},\theta ))]$$

where *B_wi_* denotes the optimal bandwidth for the *i*th segment. As the vibration signal is angular resampled, the time variable *t* is replaced by the angular variable *θ* for the convenience of explanation. According to Equation (9), the phase modulation of the *i*th segment can be obtained by subtracting the linear phase progression of *k*th meshing harmonic from the instantaneous phase of the band-pass filtered signal

(10) $${\tilde{b}}_{k}^{B_{wi}}(f_{i},\theta )=\arg z(f_{i},\theta )-(kT\theta +\Phi_{k})$$

**Figure 7:**
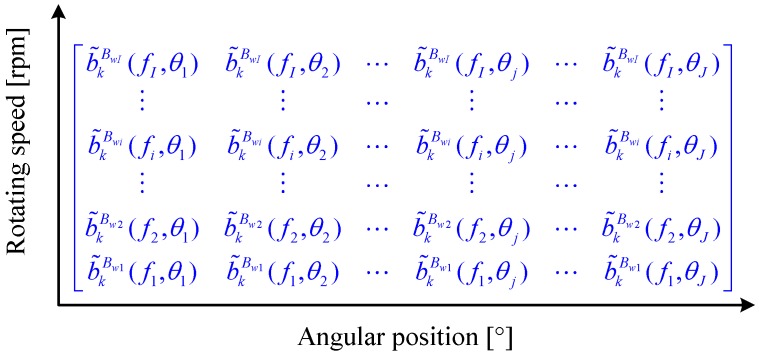
Matrix form of phasogram.

**Figure 8:**
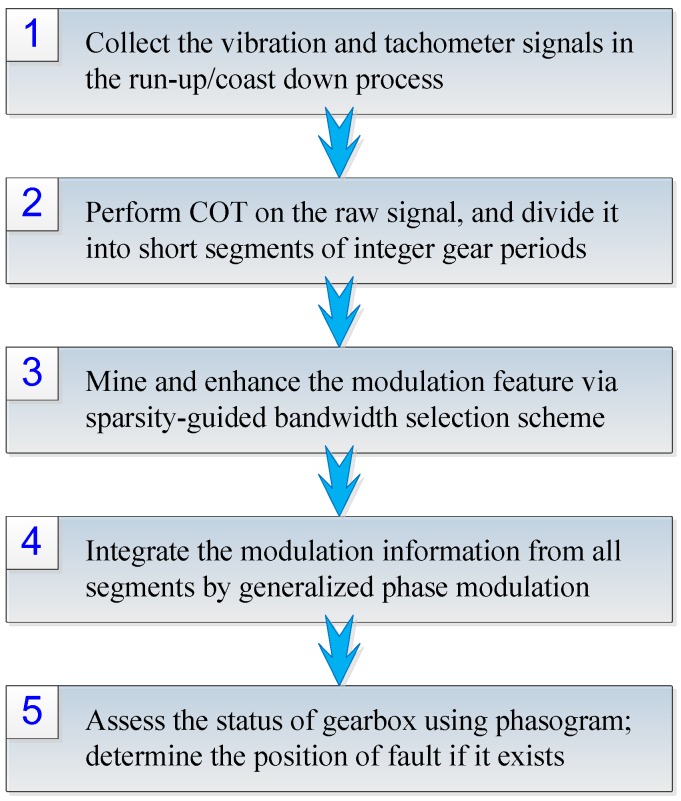
Flowchart of the proposed method.

To clearly reveal the speed-dependent modulation characteristics of gearbox, a three-dimensional representation of GPD, called phasogram, is proposed. The phasogram is created by piling ${\tilde{b}}_{k}^{B_{wi}}(f_{i},\theta )$ line by line as shown in Figure 7, with *I* denoting the number of signal segment, and *J* being the number of samples per revolution. The mathematical form of phasogram is actually a matrix of size *I* × *J*. The *x*-axis of the phasogram stands for the rotating angle of the tested gear, and the *y*-axis represents its running speed. Therefore, the element at the *i*-th row and *j*-th column of the phasogram indicates the phase modulation in position $\theta_{j}$ and at a speed of $60f_{i}$ rpm. By utilizing this representation, both the position of fault and its speed-dependent phase modulation characteristic can be recovered. To summarize, the flowchart of the proposed method is outlined in Figure 8. 

## 4. Experimental Validation

**Figure 9:**
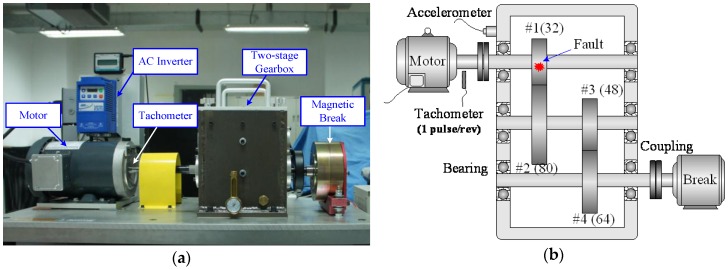
Experimental setup: (**a**) configuration of gearbox; and (**b**) schematic view.

To evaluate the performance of the proposed method, an experimental validation was conducted on a gearbox designed by the SpectraQuest, Inc. (Richmond, VA, USA). This test bed contained a two-stage spur gearbox, which was driven by an induction motor and loaded by a magnetic brake, as presented in Figure 9a. The configuration of the gearbox is illustrated in Figure 9b. The rotating speed of motor was controlled by an AC inverter, which allows the gearbox to run under a predefined speed profile. A tachometer was installed on the motor shaft to feedback the speed information to the inverter. Since the gearbox was not running under a constant speed, the characteristic frequencies of gearbox were also time-varying. For convenience, those frequencies are normalized by the motor frequency, and given in terms of order, which are listed in Table 1.

**Figure 10:**
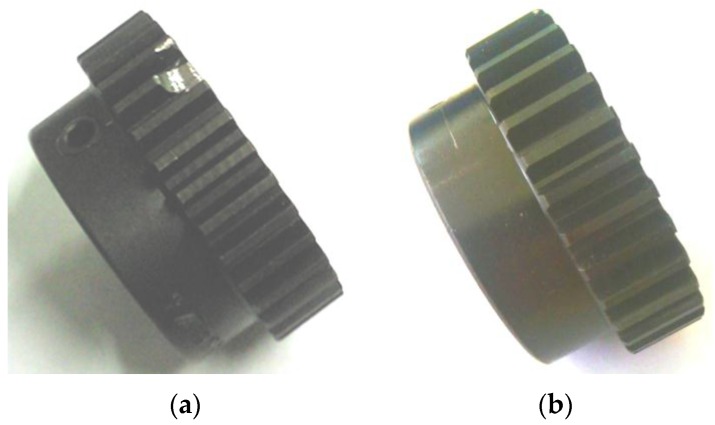
Gears used in the experiment: (**a**) chipped gear; and (**b**) normal gear.

The vibration of gearbox was measured by a piezoelectric accelerometer (PCB-356A12, PCB, Inc., New York, NY, USA), which was installed near the input shaft of gearbox. An NI data acquisition system was employed to synchronously acquire the vibration and tachometer signals, and the sampling frequency was 10,000 Hz. To simulate gear fault condition, a tooth defect was artificially introduced by chipping off a small piece with radius of 3.5 mm as illustrated in Figure 10a. This fault was located at 95° relative to the tacho-mark. For comparison, the vibration of a normal gear, as shown in Figure 10b, was also measured under the same test condition.

**Figure 11:**
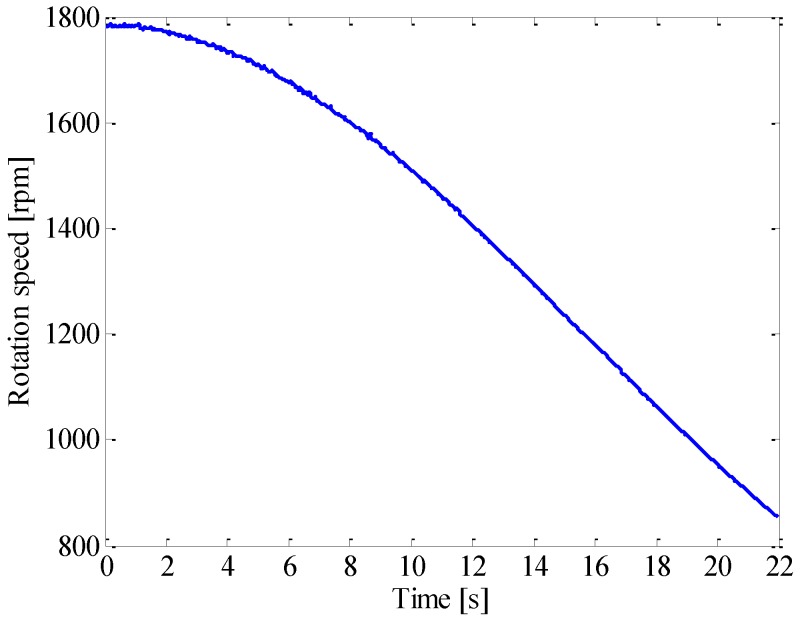
Speed profile of gearbox.

**Figure 12:**
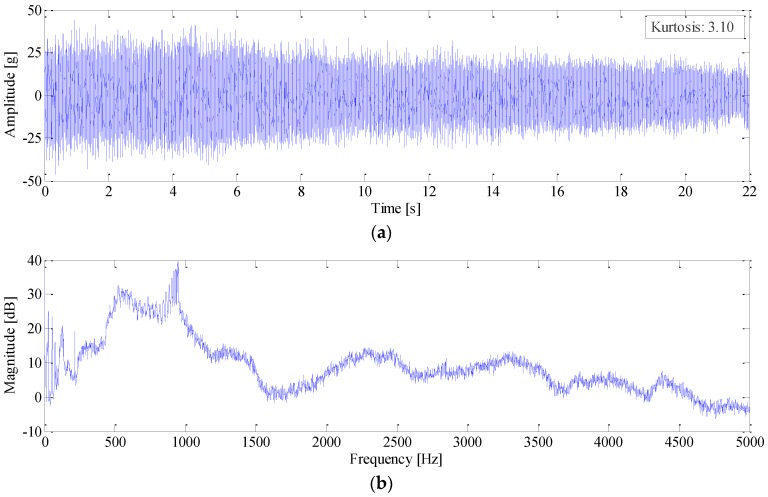
(**a**) Waveform; and (**b**) power spectrum of raw signal.

In the experiment, the gearbox went through a nonlinear coast down process as shown in Figure 11. The vibration signal of the faulty gear was measured as given in Figure 12a. It is surprising to observe that no distinct impulses can be identified in its vibration signal, and the kurtosis value is only 3.10. The reason may be attributed to the fact that, though it seems severe, the chipped tooth only partially impairs the gear tooth face as shown in Figure 10a, In fact, at least 2/3 of the tooth face is in good condition, which could sustain load in the meshing process. Therefore, this type of fault merely decreases the local contact stiffness, but would not generate large impulses since the meshing process is overall continuous. The power spectrum of the signal is presented in Figure 12b. As expected, the spectrum smears seriously due to large speed variation, thus the modulation sidebands cannot be discerned. 

**Figure 13:**
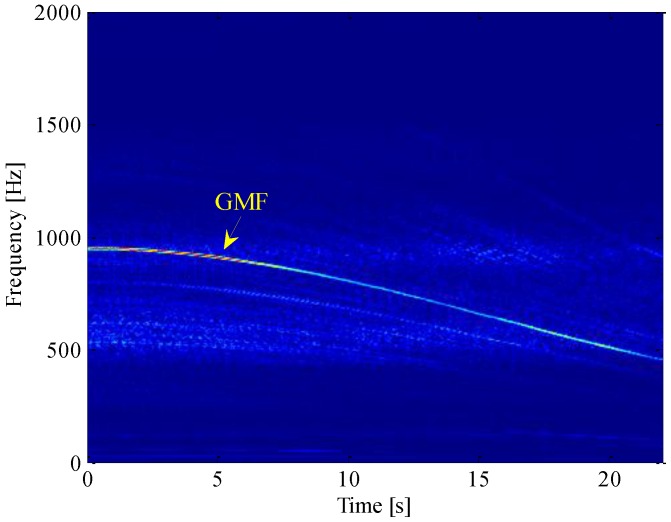
STFT of raw signal.

For more information, short-time Fourier transform (STFT) is employed to analysis the time-frequency distribution (TFD) of the vibration signal. A Hanning window of length 0.2 s is chosen as the windowing function, and its overlap ratio is specified as 50%. The STFT of the signal is shown in Figure 13. Note that there is a dominant curve in the time-frequency plane, which corresponds to the GMF of the first stage in the gearbox. However, due to the limited time-frequency resolution of STFT, it is usually difficult to precisely recover the sideband information, especially when the acceleration of gearbox is very high. To extract the fault feature, different wavelet transforms (WTs) have also been performed on this signal. Nevertheless, two practical limitations of WT are how to select a suitable mother wavelet and how to dynamically optimize its parameters, in order to better match the speed-dependent modulation structure of the R/C signal. In addition, if the signal is not properly preprocessed, the weak fault feature could easily be overwhelmed by the high energy components like GMF and its harmonics, which in turn affects the performance of WT. 

**Figure 14:**
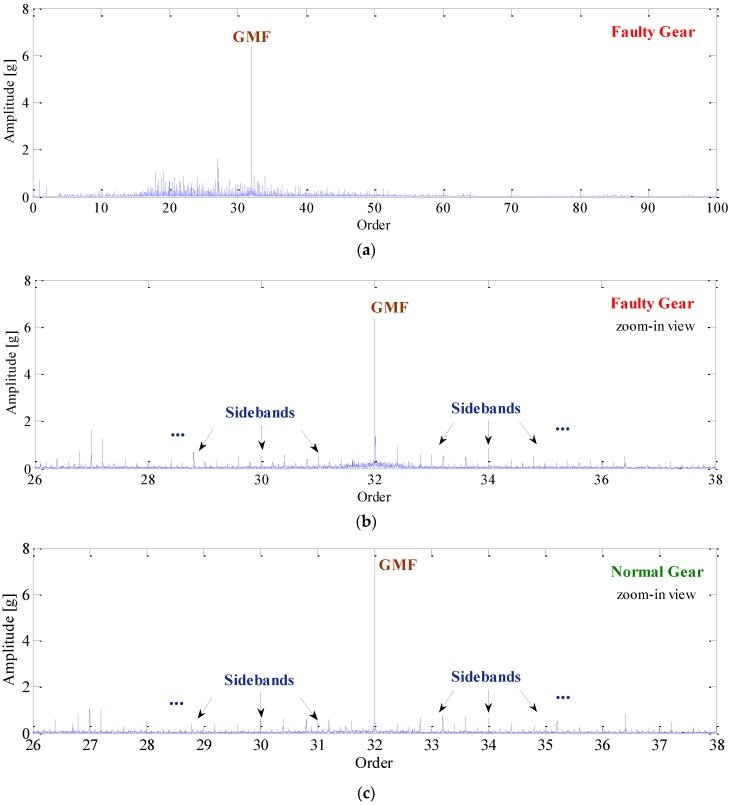
(**a**) Order spectrum of faulty gear; (**b**) zoomed-in order spectrum of faulty gear; and (**c**) zoomed-in order spectrum of normal gear.

As discussed in Section 3, it is necessary to eliminate the spectral smearing effect before the application of more advanced signal processing techniques. For this purpose, COT is performed on the raw signal by utilizing the tachometer information. The order spectrum of resampled signal is presented in Figure 14a. For more details, this spectrum is zoomed in the range of 26–38 orders as shown in Figure 14b. Note that the smearing problem is solved effectively after COT and, thus, the meshing frequency and its sidebands can be identified clearly. However, it is also found that these sidebands are very weak in amplitude, and it would be risky to use them as an indication of fault. To demonstrate this, the vibration signal of a normal gear is also analyzed and shown in Figure 14c. It is found that the sidebands also exist in the order spectrum of normal gear due to geometric errors and assembly imperfections. For this reason, it is usually difficult to identify the gear fault by using COT alone.

**Figure 15:**
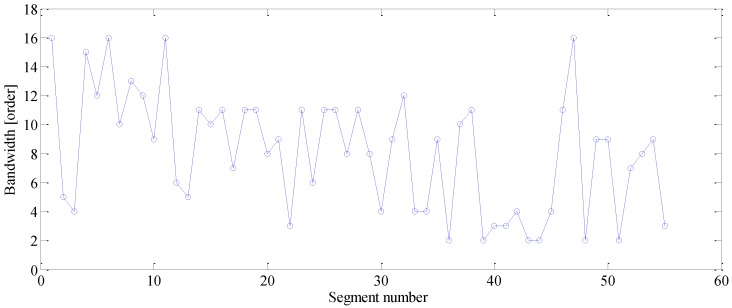
Determined bandwidth for each segment.

To exploit the more useful features embedded in the R/C process, the proposed method is applied to the same signal. Firstly, the order tracked signal is divided into short segments as discussed in Section 3. Each segment contains exactly 20 revolutions of vibration signal of Gear #1, and the sliding step is specified as 10 revolutions of that gear. To enhance the modulation feature induced by fault, the sparsity-guided phase jitter sharpening scheme is used to determine a proper demodulation bandwidth for each segment. The corresponding result is presented in Figure 15. It can be seen that the optimal bandwidths fluctuate widely over segments (with a standard deviation of 4.13 orders). For instance, the optimal bandwidth for 10th segment is nine orders, while those for 22nd and 47th are three and 16 orders, respectively. This large fluctuation implies that one can hardly find a fixed bandwidth that is well suited to all segments, which explains why a date-driven bandwidth selection scheme is essential in R/C signal analysis.

**Figure 16:**
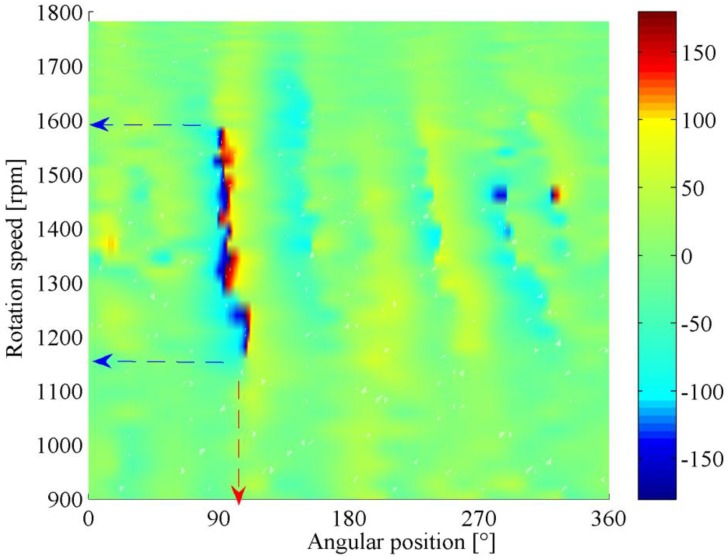
Phasogram of the faulty gear.

Following the above feature mining and enhancement procedure, the phase demodulation result for each segment is integrated by the proposed GPD, and the corresponding phasogram is presented in Figure 16. In this figure, a sharp color change from blue to red can be observed clearly, which reveals the fault-induced phase jitter buried in the vibration signal. Based on this phenomenon, one can conclude that a localized defect occurs in the gear. Moreover, the projection of the shade onto the horizontal axis could indicate the accurate position of defect, i.e., located at an angle of 95° relative to the tacho-mark, while its projection onto the vertical axis reveals the optimal test speed of gearbox, i.e., 1150–1600 rpm.

**Figure 17:**
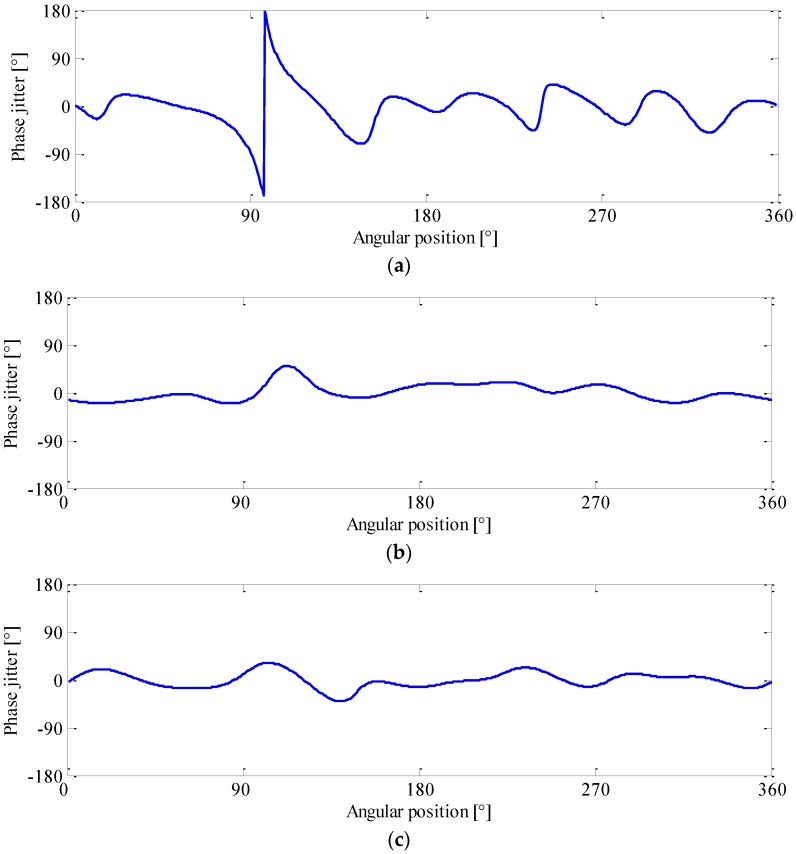
Phase demodulation results obtained at: (**a**) 1350 rpm; (**b**) 1100 rpm; and (**c**) 1650 rpm.

In Figure 16, one can also observe that the modulation feature of gearbox is speed-dependent, and the gear defect is not always detectable under arbitrary operating speed. In classical demodulation methods, the modulation information is extracted at a constant speed. Therefore, their reliability and effectiveness largely rely on the test speed selection. For example, if the defective gear is tested at a running speed of 1350 rpm, the phase jitter could be clearly recognized as presented in Figure 17a. Nevertheless, if it is measured at 1100 or 1650 rpm, no distinctive phase jitter can be identified as shown in Figure 17b,c. In these cases, the gear fault is likely to be miss-detected. As opposed to classical methods, the proposed GPD could yield a more reliable health assessment result of gearbox. Through the information mining and integration, both the location of fault and its speed-dependent modulation characteristics can be revealed comprehensively in the phasogram. 

**Figure 18:**
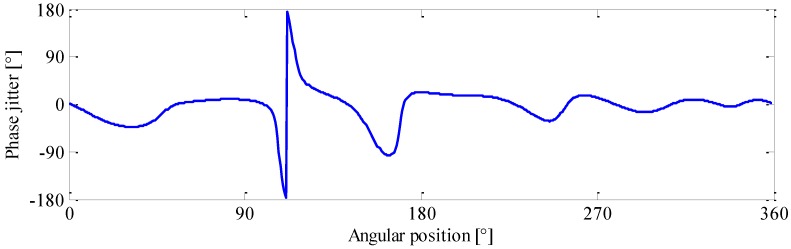
Classical phase demodulation result of the normal gear.

Another advantage of the proposed method lies in its capability in false alarm resistance. Figure 18 represents another phase demodulation result using classical method. Without prior knowledge, it is probably associated with certain gear fault. Nevertheless, this signal indeed came from a normal gear. 

**Figure 19:**
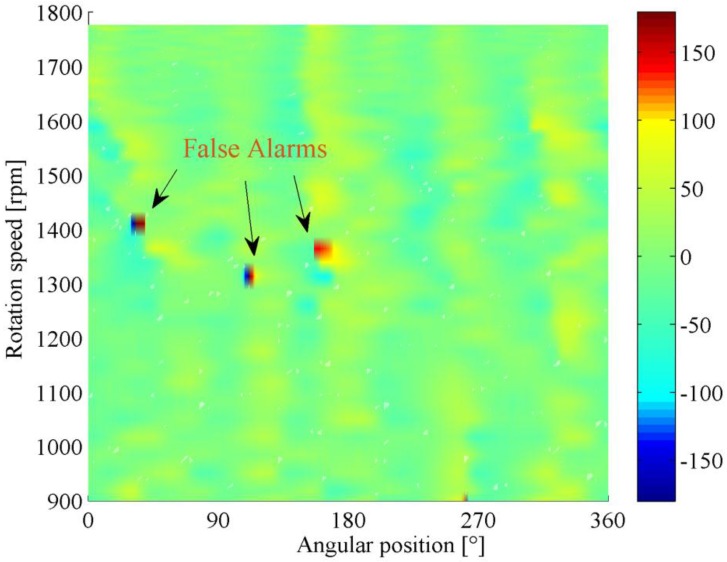
Phasogram of the normal gear.

In fact, even for the gear in healthy state, its modulated phase signal might contain some phase jitters caused by measurement noise or ambient interferences. In conventional approaches, it is not easy to determine where these phase jitters come from due to the lack of information. This drawback can be effectively overcome by the phasogram. Since the modulation features under different speeds are integrated, the false alarms can easily be eliminated by inspecting the phase modulation pattern in the phasogram. For a real fault, since its location is fixed and it is usually detectable within a certain speed interval, the corresponding phase modulation typically occupies a stripe-like region along speed axis as shown in Figure 16. In contrast, the phase modulation caused by interference is manifested as sporadic points which are randomly distributed in the phasogram as presented in Figure 19. This is determined by the stochastic behavior of noise. Hence, it can be concluded that the corresponding gear of Figure 19 is in healthy condition.

## 5. Conclusions and Future Work

This work investigates the feature mining and health assessment of gearbox using the R/C signals, and the following conclusions can be drawn.

The run up/down signals of gearbox contain rich information about the health condition of gears. However, due to the highly non-stationary characteristic, those signals are difficult to process and interprete. To solve this issue, COT is first employed to remove the spectral smearing effect, and a sparsity-guided feature enhancement scheme is then proposed to extract the weak phase jitter associated with gear defect. In order for an effective feature mining and integration under R/C, a generalized phase demodulation (GPD) technique is further established in this work. The proposed GPD can be viewed as an extension of traditional phase demodulation approaches. Since GPD is not based on the stationary assumption, it reveals the evolution of phase modulation over rotating speed clearly. Compared with classical methods, GPD could not only give clear evidence of fault, but also offer a guideline for the selection of optimal test speed. This property makes GPD an appealing tool for the health assessment and fault diagnosis of gearbox. The advantages of proposed method are manifested by the comparison with conventional techniques. The experimental results indicate that the gear fault, which may be miss-detected at a constant speed, could be effectively identified in the coast down process.

While attractive results are obtained, only the spall fault was considered in this work due to the experimental limitation. Therefore, the effectiveness of the proposed method on different gear defects still deserves further investigation in future work.

## Figures and Tables

**Table 1:** Characteristic frequencies of gearbox.

Input Shaft	Middle Shaft	Output Shaft	GMF of #1 and #2	GMF of #3 and #4
1	0.4	0.3	32	19.2
